# Seed after-ripening is a discrete developmental pathway associated with specific gene networks in Arabidopsis

**DOI:** 10.1111/j.1365-313X.2007.03331.x

**Published:** 2008-01

**Authors:** Esther Carrera, Tara Holman, Anne Medhurst, Daniela Dietrich, Steven Footitt, Frederica L Theodoulou, Michael J Holdsworth

**Affiliations:** 1 Crop Performance and Improvement Division, Rothamsted Research Harpenden, Hertfordshire AL5 2JQ, UK; 2 Department of Agricultural and Environmental Sciences, School of BioSciences, University of Nottingham Nottingham LE12 5RD, UK

**Keywords:** abscisic acid, after-ripening, Arabidopsis, dormancy, germination, transcriptome

## Abstract

After-ripening (AR) is a time and environment regulated process occurring in the dry seed, which determines the germination potential of seeds. Both metabolism and perception of the phytohormone abscisic acid (ABA) are important in the initiation and maintenance of dormancy. However, molecular mechanisms that regulate the capacity for dormancy or germination through AR are unknown. To understand the relationship between ABA and AR, we analysed genome expression in *Arabidopsis thaliana* mutants defective in seed ABA synthesis (*aba1-1*) or perception (*abi1-1*). Even though imbibed mutant seeds showed no dormancy, they exhibited changes in global gene expression resulting from dry AR that were comparable with changes occurring in wild-type (WT) seeds. Core gene sets were identified that were positively or negatively regulated by dry seed storage. Each set included a gene encoding repression or activation of ABA function (*LPP2* and *ABA1*, respectively), thereby suggesting a mechanism through which dry AR may modulate subsequent germination potential in WT seeds. Application of exogenous ABA to after-ripened WT seeds did not reimpose characteristics of freshly harvested seeds on imbibed seed gene expression patterns. It was shown that secondary dormancy states reinstate AR status-specific gene expression patterns. A model is presented that separates the action of ABA in seed dormancy from AR and dry storage regulated gene expression. These results have major implications for the study of genetic mechanisms altered in seeds as a result of crop domestication into agriculture, and for seed behaviour during dormancy cycling in natural ecosystems.

## Introduction

Seed survival following shedding from the mother plant is a key component in the life history of flowering plants. Under natural conditions, seed survival in the soil and cycling through states of dormancy are major ecological characters determining entry and persistence in ecosystems (Baskin and Baskin, 1998; Bewley and Black, 1994), and seed dormancy is a major trait altered during domestication of wild species (Harlan, 1992). Embryo dormancy is a genetically and environmentally determined developmental state imposed following imbibition of mature seeds, in which cell metabolism is active, but growth processes are repressed (Bewley, 1997). The potential for embryo dormancy is overcome through the time- and environment-sensitive process of after-ripening (AR) that occurs in the dry seed. Germination proceeds through three phases of water uptake: non-dormant seeds successfully complete the transition from phase two to three (radicle elongation), whereas dormant seeds remain within phase two (Bewley and Black, 1994). Following imbibition, the AR status of the seed determines whether individual seeds complete germination or remain dormant. AR therefore represents a key characteristic of seeds in the determination of germination potential, which can be defined as the capacity of seeds to complete germination once imbibed. A recent definition of AR suggested that it is a widening or increased sensitivity of perception of seeds to environmental conditions promoting germination, at the same time as a narrowing or decrease in sensitivity of perception of conditions repressing germination (Finch-Savage and Leubner-Metzger, 2006). Thus, environmental conditions that favour germination will not trigger germination at early stages of AR, but may do so following the appropriate AR time. Despite a huge literature describing the physiological aspects of AR, nothing is known about the molecular mechanisms that control AR in the dry seed or about the downstream regulation of gene expression by AR in the imbibed seed (Finch-Savage and Leubner-Metzger, 2006; Kucera *et al.*, 2005).

Measurement of water contents of unimbibed seeds suggests that cellular environments are compatible with the operation of complex biochemical processes, and that AR rate is affected by seed water content (Finch-Savage and Leubner-Metzger, 2006; Foley, 1994; Manz *et al.*, 2005). Biochemical processes can occur in the unimbibed seed during storage, including changes in metabolites (Bailly, 2004; Clerkx *et al.*, 2004), proteome (Chibani *et al.*, 2006) and gene expression (Bove *et al.*, 2005; Leubner-Metzger, 2005), providing possible mechanisms through which the AR process may work.

A large number of loci that regulate germination potential have been identified through genetic studies in *Arabidopsis thaliana*. Mutation of these loci range from subtle changes in germination potential and dormancy capacity, to extreme conversion of embryo characteristics into those of seedlings, as in severe *abi3*, *lec* and *fus3* mutants (Holdsworth *et al.*, 1999). Many loci are involved in synthesis or signal transduction of phytohormones, principally gibberellins, ethylene and abscisic acid (ABA; Finch-Savage and Leubner-Metzger, 2006). Genetic evidence shows that synthesis of ABA during late stages of embryogenesis is required for dormancy (Karssen *et al.*, 1983). Arabidopsis mutant *aba1* seeds, which are deficient in zeaxanthin epoxidase, the first enzyme in ABA-specific biosynthesis (Marin *et al.*, 1996), are non-dormant (Koornneef *et al.*, 1982). Recently, it was shown that mutant seeds deficient in ABA 8′-hydroxylase, which catabolizes ABA, show increased dormancy levels, suggesting that ABA breakdown is required to remove dormancy (Kushiro *et al.*, 2004). Signal transduction following ABA synthesis is also important for the repression of germination, and mutants have been identified in Arabidopsis that are either insensitive (e.g. *abscisic acid insensitive1*, *abi1*) or hypersensitive (e.g. *lipid phosphate phosphatase2, lpp2*) in their germination response to applied ABA (Katagiri *et al.*, 2005; Koornneef *et al.*, 1984). A general property of AR seeds is lowered levels of, and decreased sensitivity to, ABA compared with freshly harvested seeds (Ali-Rachedi *et al.*, 2004; Grappin *et al.*, 2000). Application of exogenous ABA has previously been used as an experimental manipulation to mimic dormancy in AR seeds, and it has been shown that ABA added to imbibed AR seeds delays endosperm rupture in Arabidopsis (Müller *et al.*, 2006). However, ABA-treated seeds may not be developmentally equivalent to dormant seeds, and studies have shown that these seeds still carry out metabolic processes associated with germination, including fatty acid breakdown (Pritchard *et al.*, 2002).

In this study we have used a transcriptome profiling approach to analyse AR as a specific developmental process. We have successfully used mutant Arabidopsis seeds to uncouple AR and germination potential by identifying AR-associated and AR-independent sets of genes. The behaviour of these gene sets defines AR as a specific developmental pathway that is separate from germination potential. These observations have implications for the study of genetic mechanisms regulating dormancy in cultivated crops, and for processes controlling seed behaviour during dormancy cycling in natural ecosystems.

## Results

### Theoretical considerations: defining AR-regulated gene sets

Previous studies have defined AR as a widening or increased sensitivity of perception of seeds to environmental conditions promoting germination, at the same time as a narrowing or decrease in sensitivity of perception of conditions repressing germination (Finch-Savage and Leubner-Metzger, 2006). This definition ties the function of dry AR (occurring in the dry seed) to the physiological result of AR (which occurs in the imbibed seed). This makes it impossible to detect changes in imbibed wild-type (WT) seeds resulting from AR-specific functions (rather than those changes that are purely a consequence of being in a physiological state, such as ‘dormant’ or ‘germinating’). We searched for experimental tools to analyse AR *per se*. Within this study we define ‘germination potential’ as the capacity of seeds to complete germination once imbibed. We reasoned that, in artificially-induced mutants with increased germination potential (that lack dormancy), these processes may be uncoupled, i.e. changes in the dry seed that would lead to the promotion of germination (AR) in WT seeds may still occur, even though mutant seeds have no dormancy. The advantage of this as an experimental tool would be that dry-stored mutant seeds, although exhibiting the same germination potential as freshly harvested mutant seeds, would have gone through the dry AR process. In this way, AR-specific gene sets could be identified as being shared between mutant and WT imbibed seeds either before or following dry storage. Because ABA has been shown to have a profound effect on germination potential, we chose to address this possibility using two mutants with impaired synthesis (*aba1-1*) or perception (*abi1-1*) of ABA, which have been shown to have constitutive effects and not to be restricted to a specific stage of development.

### Analysis of dormancy, AR and germination potential of Arabidopsis seeds

**Figure 1:**
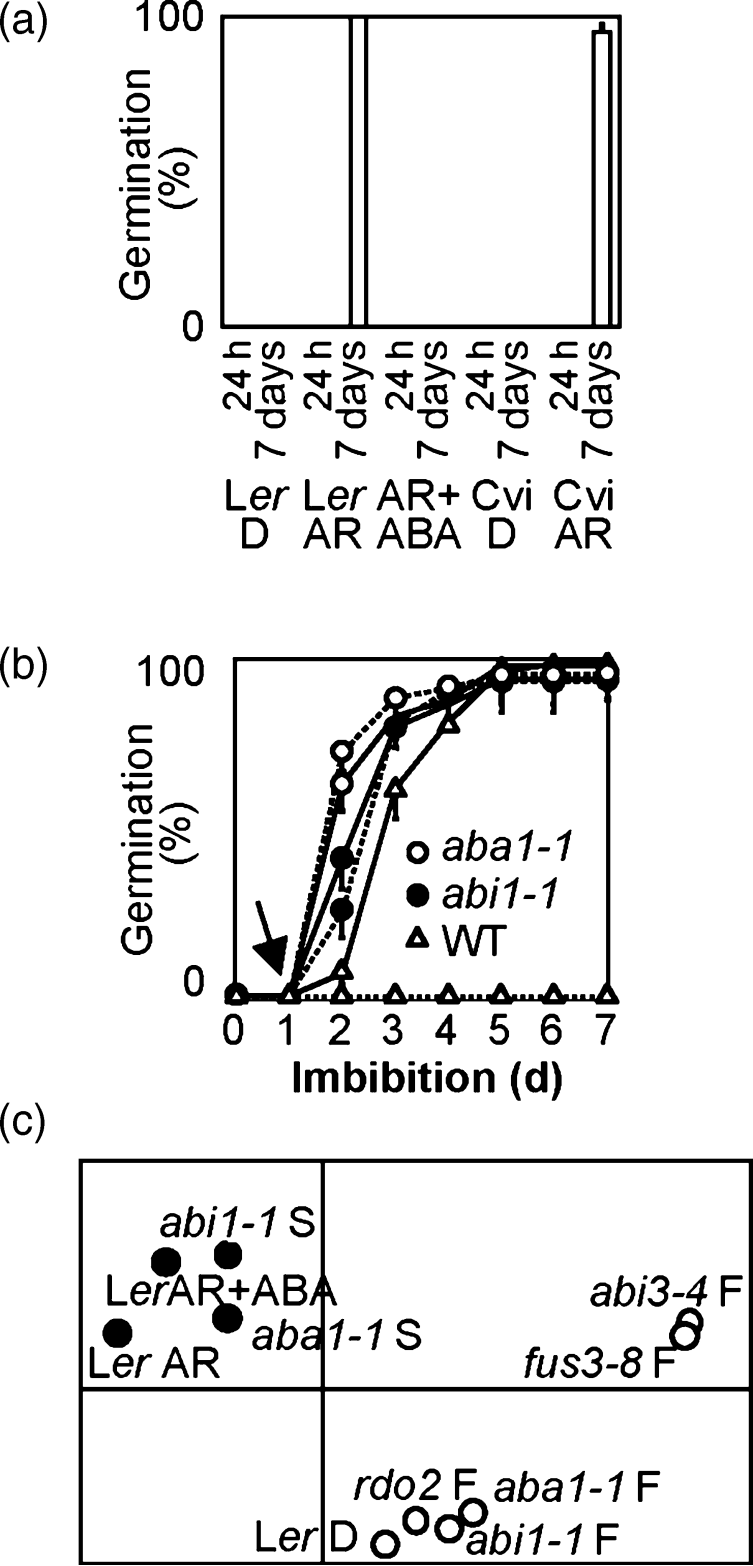
Germination potential and genome expression of wild-type (WT) and *aba1-1* and *abi1-1* mutant seeds used in this study. (a) Germination potential of WT (L*er* and Cvi) seeds at 24 h and 7 days of imbibition on water agarose in constant light [L*er* after-ripened (AR) seeds imbibed in the presence of 10 μm ABA are denoted as AR + ABA]. (b) Germination velocity of freshly harvested and stored WT and mutant seeds. The time point used for transcriptome analysis is indicated with an arrow. Freshly harvested seeds, dashed line; stored seeds, solid line. (c) Principal component analysis of transcriptome datasets derived from RNA extracted at 24 h of imbibition. Closed circles indicate samples derived from stored seed lots; open circles indicate samples derived from freshly harvested (un-stored) seed lots. AR, after-ripened; D, dormant; F, freshly harvested; S, stored seeds. The first two components accounted for 57% of the variance (35% for the first and 22%, respectively).

Seeds of WT Arabidopsis accessions, and mutants *aba1-1* and *abi1-1*, were compared for AR capacity and germination potential (Figure 1). Dormancy is removed after appropriate times of storage of WT dry seeds, after which imbibed seed lots complete germination (Alonso-Blanco *et al.*, 2003; Bentsink *et al.*, 2006) (Figure 1a, accessions L*er* and Cvi). Both *aba1-1* and *abi1-1* freshly harvested (F) seeds were able to complete germination (Figure 1b). Analysis of imbibed seeds over 7 days showed that the velocity of germination was not appreciably different between F and stored (S) mutant seeds, or between AR WT seeds and F or S mutant seeds (Figure 1b). In all cases, no germination was evident at 24 h of imbibition. Therefore at the physiological level, the germination potential of mutant F seeds is indistinguishable from AR WT seeds. However, as discussed above, it is possible that AR could still occur if it were a discrete developmental process, uncoupled from germination potential. Mutant *aba1* and *abi1* seed therefore provide material to address this question.

### Identification of AR-associated and AR-independent gene sets in imbibed seeds

We used transcript profiling to define AR-related gene sets and to determine whether transcriptional events in imbibed seeds associated with AR still occur in mutants with altered germination potential. RNA samples for transcriptome analysis were derived from highly synchronous WT or mutant seed populations with controlled environment histories, and whole genome expression was analysed using the ATH1 Affymetrix Genechip. Transcriptomes of seeds were assayed at 24 h of imbibition, because this captures gene expression in phase II of germination (prior to radicle elongation, Figure 1), which results from the AR status of the dry seed. Sets of genes that showed enhanced expression in specific developmental and mutant backgrounds were identified (Table S1). Whereas for most comparisons 500–1700 genes were differentially regulated, for both *aba1* and *abi1* F seeds very few genes were differentially regulated in comparison with L*er* dormant (D) seeds, suggesting that these seeds are similar at the transcriptome level. Conversely, comparisons of *aba1* and *abi1* F and S samples showed these to be very different, suggesting that changes occur in these non-dormant mutant seeds under the same conditions that promote AR in D seeds, and therefore that AR may occur in these non-dormant mutant seeds. This observation was confirmed through principal component analysis (PCA) of the datasets (Figure 1c), which showed that mutant ‘S’ and WT AR transcriptomes cluster closely, as do mutant ‘F’ and WT D transcriptomes. Using this approach, we were also able to show that the transcriptome of the F imbibed non-dormant *rdo2* mutant (Léon-Kloosterziel *et al.*, 1996) also clustered with L*er* D. Non-dormant *abi3-4* and *fus3-8* seeds are physiologically unlike *aba1-1* and *abi1-1* in that they demonstrate post-germination seedling characteristics at the end of embryo maturation (including vascularization and apical meristem activation) (Holdsworth *et al.*, 1999). Accordingly, PCA of transcriptomes from F *abi3-4* and *fus3-8* seeds clustered together, and far from other F, D or S AR samples.

**Figure 2:**
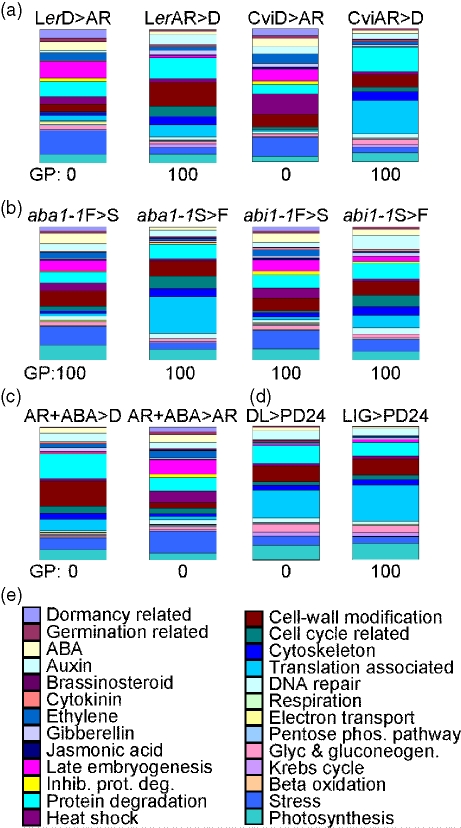
Definition of ‘developmental signatures’ associated with differentially regulated gene sets in 24-h imbibed seeds. For each comparison the proportional representation of genes identified by the TAGGIT workflow is shown. Datasets are derived from Table S1; in each case ‘>’ indicates the upregulated gene set in the comparison. For example; F > S means that the genes presented are upregulated in the fresh (F) compared with the stored (S) state, i.e. F upregulated gene set. Germination potential of seeds (%) for the upregulated comparator is shown under each signature diagram (GP). (a) Comparisons of L*er* and Cvi wild-type (WT) seeds in different developmental states (after-ripened, AR; dormant, D). (b) Comparisons of *aba1-1* and *abi1-1* mutant seeds either freshly harvested (F) or stored (S) for a length of time that allowed complete AR of WT L*er* seeds. (c) Comparisons of L*er* AR seeds imbibed either with or without 10 μm ABA. (d) Comparisons of Cvi WT primary dormant 24-h (PD24) AR imbibed in the dark (DL), and AR imbibed in the dark for 20 h and then given a 4-h red light pulse (LIG), seeds. Data in panel (d) derived by reanalysis of the transcriptome datasets of Cadman *et al.* (2006). (e) Key showing colours relating to functional groups.

To distinguish cohorts of genes encoding proteins related to biological processes associated with dormancy and germination, we analysed lists of differentially expressed genes using a novel approach that re-annotates gene lists according to defined functions. This approach, using the TAGGIT workflow, replaces the commonly used gene ontology (GO) annotation, because it provides more useful information related to seed biology (Carrera *et al.*, 2007). This provides a ‘developmental signature’, tagging that part of the transcriptome with known functions in relation to dormancy and germination (Figure 2). This analysis showed clear differences between D or AR upregulated genes in both L*er* and Cvi WT accessions, and demonstrated that characteristic ‘D’ and ‘AR’ signatures were easily distinguishable (Figure 2a, derived from Table S1). Using this approach, it was possible to show that signatures of genes upregulated in F compared with S seeds (F > S) of both *aba1* and *abi1* more closely resembled those of the WT D > AR differentially upregulated gene group, and that signatures of *aba1* and *abi1* S > F resembled those of WT AR > D (Figure 2b).

**Figure 3:**
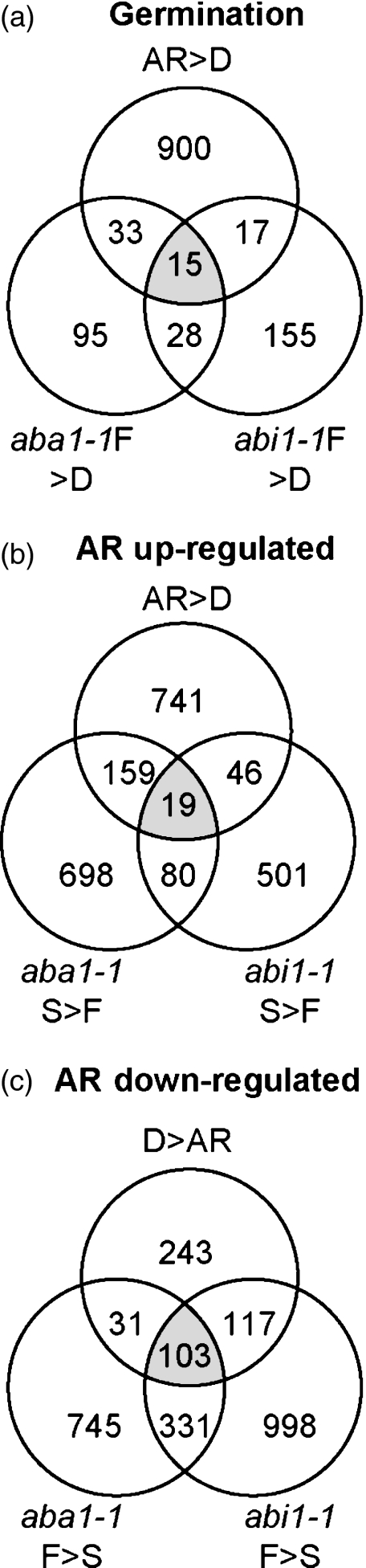
Venn diagram representations identifying after-ripening (AR)-regulated and AR-independent gene sets in 24-h imbibed seeds. Datasets are derived from Table S1; in each case ‘>’ indicates the upregulated gene set in the comparison. In each case sets used for comparison are indicated next to associated circles. The numbers of genes represented in the gene sets are shown within the intersecting and non-intersecting segments of the sets. Gene lists for all comparisons are presented in Table S2a,b. In all cases AR and dormant (D) refer to datasets of L*er* wild-type seeds. (a) Identification of a gene set associated solely with germination potential in imbibed seeds (‘germination’ upregulated). (b) Identification of a gene set associated solely with stored (S)/AR imbibed seeds. (c) Identification of a gene set associated solely with fresh (F)/non-AR (dormant) imbibed seeds. A box-plot analysis showing statistical data associated with the different gene groups is given in Figure S1.

Cohorts of genes that were AR-associated or expressed independently of AR were obtained from comparisons of differentially regulated genes (Figure 3). Comparison of genes from F, D or S AR differentially expressed gene sets (Table S1) identified 15 of which expression was AR-independent and associated solely with germination potential (‘Germination’, Figure 3a; Table S2), 19 genes common to all S-AR sets (AR upregulated, Figure 3b, which includes the gene *LPP2* that removes sensitivity to ABA during germination) and 103 common to F-D sets (AR downregulated, Figure 3c, which includes *ABA1*, involved in ABA synthesis). The expression patterns of representative members of each set was measured in WT and mutant seeds from 0–48 h of imbibition using quantitative (Q)RT-PCR (Figure S2). For each gene, similar biases in expression in F and S seeds were seen to those observed in transcriptome experiments.

**Figure 4:**
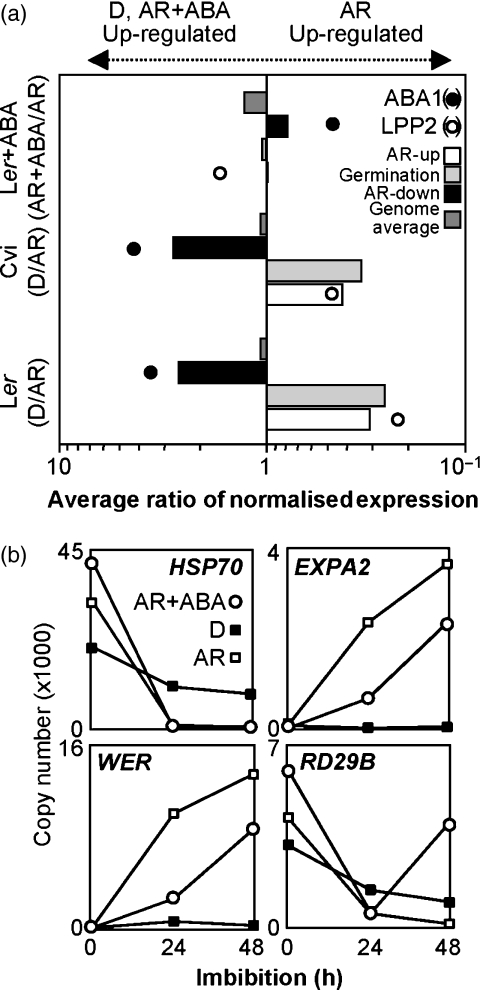
Comparison of wild-type accessions and influence of exogenous ABA on expression characteristics of after-ripening (AR)-associated and AR-independent gene sets at 24-h imbibition. (a) Average ratio of expression of the ‘AR-upregulated’, ‘AR-downregulated’ and ‘germination-upregulated’ gene sets in L*er* and Cvi. In each case ratio components are indicated [for example L*er*(D/AR) indicates the average ratio of expression of genes from accession L*er* using dormant and AR samples]. Normalized expression values and ratio averages are for gene sets derived from microarray datasets (Table S3). Ratio values for *ABA1* and *LPP2* are plotted. A more complete box-plot analysis showing statistical data associated with means is given in Figure S4. (b) Quantitative rtPCR time-course analysis of expression of representative members of the ‘AR-upregulated’ (*WER*), ‘AR-downregulated’ (*HSP70B*, *RD29B*) and ‘germination-upregulated’ (*EXPA2*) gene sets over 48 h of imbibition on water agarose in the absence (AR, D) or presence (+ABA) of 10 μm ABA in constant light. D, dormant seeds; AR, after-ripened seeds.

In order to further examine the robustness of expression characteristics for these gene sets, we also analysed global gene expression in WT accession Cvi. Comparison of transcriptomes of AR and D Cvi seeds showed large changes in expression between the two physiological states at 24 h of imbibition (Table S1). To compare expression properties of the AR-associated and AR-independent gene sets, we derived the average ratio of expression levels of each gene set, comparing either different physiological states or different genetic backgrounds (Figure 4a, Figure S4). Comparison of the D with the AR state in the WT accessions L*er* and Cvi revealed very similar average ratios of expression of each gene set. Ratios of expression for the genes *ABA1* and *LPP2* were consistent with their placement in ‘AR-downregulated’ and ‘AR-upregulated’ sets, respectively.

### Influence of exogenous ABA

Treatment of AR WT seeds with exogenous ABA greatly reduced germination potential over the time period taken by untreated seeds to complete germination (Figure 1a) (although ABA-treated seeds do eventually germinate; Müller *et al.*, 2006). However, if AR and dormancy are independent developmental processes, manipulation of the germination potential of AR seeds by application of ABA should not influence AR-related gene expression. Transcriptome analysis showed that many genes were differentially regulated between ABA-treated and untreated WT AR seeds at 24 h of imbibition in comparison with WT D and AR seeds (Table S1). Twice as many genes were differentially expressed when comparing ABA-treated AR seeds with D seeds, than when comparing AR ABA-treated seeds with untreated AR seeds, suggesting that ABA-treated AR seeds more closely resemble AR seeds than D imbibed seeds. PCA analysis demonstrated that the transcriptome of ABA-treated AR seeds more closely resembled untreated AR than D seeds (Figure 1c). Assay of functional representation by developmental signature analysis demonstrated that the (AR + ABA) > D differentially regulated gene set resembled the AR > D set, including augmented representation of genes associated with protein degradation, cell cycle and translation (Figure 2c). In comparison, the signature of (AR + ABA) > AR set more closely resembled the D > AR set, suggesting that ABA does induce transcripts associated with dormancy, although less than 10% of specific genes were shared. Analysis of the average ratios of expression of the three gene sets demonstrated that, in comparison with expression in the D state, addition of ABA to AR seeds did not significantly alter expression of any of the gene sets (Figure 4a). Quantitative RT-PCR examining the dynamics of expression of members of these gene sets confirmed that ABA treatment of AR seeds did not result in D-like expression (Figure 4b). Expression of *RD29B* (AR downregulated) was upregulated by ABA at 48 h of imbibition, as has previously been shown for this ABA-inducible gene (Uno *et al.*, 2000).

### Influence of light environment on gene expression in AR seeds

**Figure 5:**
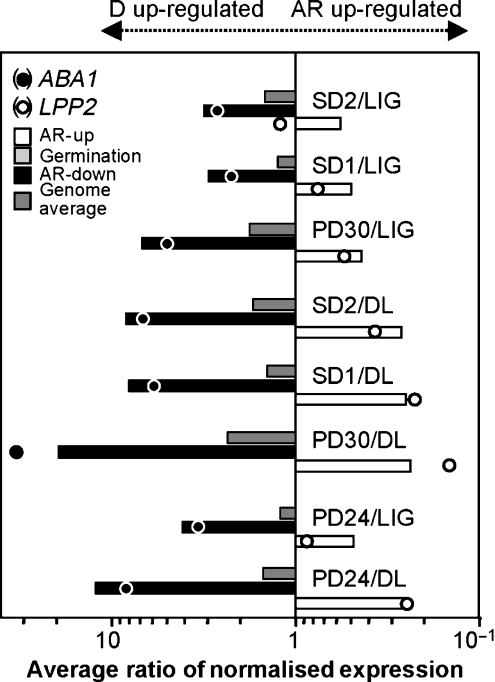
Influence of primary and secondary dormancy status on expression characteristics of after-ripening (AR)-regulated gene sets at 24 h of imbibition. Average ratio of expression of the ‘AR-upregulated’ and ‘AR-downregulated’ gene sets in Cvi are shown (Cadman *et al.*, 2006). In each case ratio components are indicated. Normalized expression values and ratio averages for gene sets derived from microarray datasets (Table S3). Ratio values for *ABA1* and *LPP2* are plotted. A more complete box-plot analysis showing statistical data associated with means is given in Figure S4. For each gene set, data are presented for ratios for the following datasets: primary dormant 24-h imbibed (PD24), primary dormant 30 days imbibed (PD30), AR seeds imbibed in the dark (non-germinating, DL), AR seeds imbibed in the dark given a flash of red light to induce germination (LIG), secondary dormancy state 1 (SD1), secondary dormancy state 2 (SD2). AR, after-ripened; D, dormant; F, freshly harvested; S, stored seeds.

In addition to effects of exogenous application of hormones, environmental conditions of the imbibed seed, in particular light, can radically influence germination potential, but would not be expected to influence AR-associated gene expression, as this is a post-imbibition associated phenomenon. Treatment with red light is a common mechanism for breaking some types of dormancy (Bewley and Black, 1994). Cadman *et al.* (2006) showed that imbibed AR Arabidopsis Cvi accession seeds would not germinate in the dark (DL, light requiring), but would do so following a 4-h red light treatment (LIG, light induced to germinate). Therefore, DL and LIG seeds have completed AR, but have opposite germination potential. We used the transcriptome datasets of Cadman *et al.* (2006)to analyse the behaviour of the gene sets identified here. As both studies assayed transcriptomes of unchilled seeds imbibed on water–agarose at 24 h (mid phase II), direct comparison of datasets was possible. Analysis of developmental signatures showed that the LIG > PD24 (PD24, 24-h imbibed primary D seeds) and DL > PD24 differentially regulated gene sets were highly similar (Figure 2d), and virtually indistinguishable from the CviAR > D set (Figure 2a), despite exhibiting opposite germination potential. In addition, average ratios of expression of ‘AR-upregulated’ and ‘AR-downregulated’ gene sets were similar in both PD24/DL and PD24/LIG comparisons (Figure 5).

### Influence of secondary dormancy on AR-regulated gene expression

Manipulation of the environment of imbibed AR seeds can lead to the induction of secondary dormancy (Baskin and Baskin, 1998; Bewley and Black, 1994). Induction of secondary dormancy would be predicted to reset the expression characteristics of AR-regulated genes, because in the absence of dormancy breakage in the imbibed state, these seeds would require dry AR to complete germination. The transcriptomes of two secondary dormant states in Cvi have been reported by Cadman *et al.* (2006). We analysed these datasets to test whether the expression characteristics of AR gene sets identified in the present study are indeed altered by secondary dormancy states (Figure 5). Expression in different D states (30 days imbibed primary D seeds, PD30; secondary dormancy states 1 and 2, SD1 and SD2) was compared with expression in the two physiologically-different AR states (DL and LIG). Analysis of the average ratio of expression of each gene set showed that, for the ‘AR-upregulated’ set, expression was reduced in D seeds, whereas expression of the ‘AR-downregulated’ set was enhanced (Figure 5, Figure S4b). This shows that gene sets behaved in a similar way in all physiological states. These results show that, following AR of the dry seed, the expression of these gene sets can be ‘reset’ if the SD status of the imbibed seed is changed. Analysis of ratios of expression of *ABA1* and *LPP2* showed that these maintained an AR-related pattern.

## Discussion

**Figure 6:**
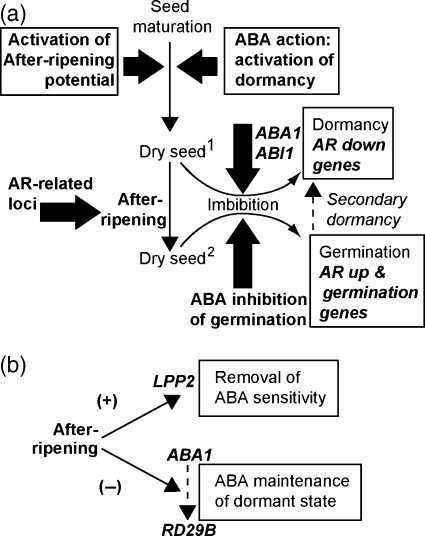
A model describing the relationships between after-ripening (AR)-regulated genes, AR as a distinct developmental process, and the role of exogenous and endogenous ABA in the regulation of the seed transcriptome. (a) Thin arrows indicate developmental processes; thick arrows indicate the influence of specific factors on developmental pathways. Superscript numbers indicate pre (1) and post (2) AR states of unimbibed seeds. A dotted line is used to indicate imbibed seeds induced into a secondary dormancy state. Prior to seed desiccation both AR and dormancy (D) are activated, dormancy by ABA action, AR by an independent unknown mechanism. Following desiccation unimbibed seeds go through a process of dry AR. Freshly harvested imbibed seeds are dormant through the action of ABA, and ‘AR-downregulated’ genes are expressed. Following AR imbibed seeds express ‘AR-upregulated’ and ‘germination-upregulated’ genes. Exogenously applied ABA can inhibit the completion of germination in AR seeds, but does not alter the expression of these gene sets, or reimpose expression of ‘AR-downregulated’ genes. Imbibed AR seeds can be reintroduced into a secondary D state, which reimposes upregulation of ‘AR-downregulated’ gene set expression and downregulation of ‘AR-upregulated’ gene set expression. (b) The relationship between AR and two genes regulating ABA synthesis (*ABA1*) or perception (*LPP2*). *ABA1* is a member of the ‘AR-downregulated’ gene set, and itself regulates 121 genes (differentially regulated in the D > *aba1-1* F comparison, Table S1), and *LPP2* is a member of the ‘AR-upregulated’ gene set, which has previously been shown to reduce sensitivity of the seed to ABA (Katagiri *et al.*, 2005). *RD29B* is a member of the ‘AR-downregulated’ gene set, itself regulated by ABA (Figure 4b).

At present, little is known about the molecular mechanisms that regulate the process of AR, or about possible downstream target genes in imbibed seeds. More generally, nothing is known about AR as a discrete process in isolation from dormancy and germination potential (Finch-Savage and Leubner-Metzger, 2006). In this paper we used a targeted transcriptomics approach to define dry seed AR as a distinct developmental process, and to identify gene sets that were associated with AR or in which expression was dependent only on germination potential. A model describing the regulation of AR, AR-regulated gene expression and the relationship of AR to ABA is presented (Figure 6a,b).

Expression characteristics of gene sets have allowed us to make and test specific predictions relating to the regulation of AR, and the relationship of AR to seed ABA biology. One important feature of AR-regulated gene expression is that it should not simply be related to germination potential. Using *aba1-1* and *abi1-1* mutant seeds, which have a constant high germination potential regardless of dry seed AR time, we showed that genome expression in imbibed seeds changed with dry seed storage time. PCA and analysis of developmental signatures both indicated that gene expression changes resulting from dry storage in each mutant were similar to those in WT seeds, suggesting a common cohort of AR-regulated genes in imbibed seeds. Three categories of gene sets were defined: those that showed positive or negative AR-associated expression, and those in which expression appeared to be independent of AR but dependent on germination potential. Exogenously applied ABA greatly reduced germination potential of AR WT seeds, but did not appreciably alter expression of AR-related gene sets. However, 80% of the germination-associated gene set was present in the AR > AR + ABA list, demonstrating the dependence of this gene set on germination. In particular, the ‘AR-downregulated’ set was not induced in ABA-treated AR seeds, even though a developmental signature similar to that of D seeds was evident. This indicates that ABA-treated AR seeds are in a different physiological state to D seeds (Figure 6). Previous work has suggested that metabolic (Pritchard *et al.*, 2002) and physiological (Müller *et al.*, 2006) processes in ABA-treated seeds are not equivalent to those of D seeds, and therefore we conclude that AR seeds treated with exogenous ABA should not be used as a method to study the relationship between ABA biology and dormancy or AR. The observation that *aba1-1* and *abi1-1* mutant seeds do after-ripen demonstrates that AR is a process that can occur independently of ABA (Figure 6a), although this does not rule out the existence of a parallel ABA-dependent pathway. Two recent studies also defined D- and AR-related gene sets, based on expression profiles of genes in Arabidopsis WT accession Cvi subjected to different environmental stimuli (Cadman *et al.*, 2006; Finch-Savage *et al.*, 2007). Those studies are different from that presented here, as manipulation of WT dormancy levels does not divorce AR from the potential of the seeds to germinate. Nevertheless, approximately half of the ‘AR-downregulated’ gene set are represented in the D-related gene sets, and one third of the ‘AR-upregulated’ gene set are represented in the AR-related gene sets, respectively (data not shown).

Our hypothesis predicts that subjecting AR seeds to environmental conditions that alter germination potential should not influence the expression of AR-related gene sets. In agreement with this, analysis of the transcriptome datasets of Cadman *et al.* (2006) using the TAGGIT methodology showed that the AR-associated gene sets maintained predictable expression patterns, independent of germination potential (Figure 6). It is also predicted that imposition of secondary dormancy should ‘reimpose’ an AR requirement on unimbibed seeds, and thereby ‘reset’ expression of these gene sets. Analysis of the transcriptome datasets of Cadman *et al.* (2006) showed that this was the case (Figure 5), and induction of two secondary dormancy states led to the restoration of AR-related gene expression characteristics (i.e. reduced expression for the ‘AR-upregulated’ set, and enhanced expression for the ‘AR-downregulated’ set).

As our approach focused on only one time point of analysis, and one approach to the identification of AR-regulated genes, it is likely that the gene sets identified here are only subsets of potential AR-associated genes. In addition, subtle differences in timing of germination/imbibition would alter observed gene expression levels at the one time point assayed, and this may partially explain why only a few genes are represented in the AR-related gene sets, and many others are excluded (Figure 3). The observation that expression of many genes conforms to an AR-regulated expression pattern provides the conceptual basis to design molecular tools with which to dissect the AR process, independent of the effects of D or germination potential. Several reports have defined quantitative trait loci (QTL) in Arabidopsis that may be candidates for loci that regulate AR (Alonso-Blanco *et al.*, 2003; Bentsink *et al.*, 2006), and introgression of these QTLs into suitable mutant backgrounds (such as *aba1* and *abi1*) may provide materials to analyse the molecular mechanisms regulating AR. It is also important to note that transcriptional changes have also been observed in ‘dry’ seeds (Bove *et al.*, 2005; Leubner-Metzger, 2005). The changes observed here in imbibed seeds at 24 h do not necessarily reflect changes in gene expression in dry seeds over storage. More analysis is required to understand the relationships between changes in gene expression in dry and imbibed seed states.

Using previously published datasets it was possible to determine several additional expression properties of genes within sets, including tissue localization within the seed. Members of the ‘AR-upregulated’ gene set showed enhanced expression in the embryo, whereas members of the ‘AR-downregulated’ and AR-independent ‘germination’ gene sets showed enhanced expression in the endosperm (Figure S3; Penfield *et al.*, 2006). These observations may indicate different functions of these subcomponents of the seed in relation to the regulation of AR or germination potential-responsive genes.

The behaviour of the different gene functional classes observed in WT and mutant seeds in response to AR agree with previously published seed transcriptomics studies (Cadman *et al.*, 2006; Ogawa *et al.*, 2003). For example, before AR, many genes associated with late embryogenesis (including heat-shock proteins and storage proteins) are evident, whereas afterwards genes associated with functions including RNA translation, cell-wall modification and protein degradation predominate (Figure 2a). Although our data indicate that AR can occur independently of ABA, they are nevertheless consistent with a role for ABA in AR-mediated processes, as the ‘AR downregulated’ and ‘AR upregulated’ gene sets included members known to control mature seed dormancy and ABA responsiveness. Specifically, the ‘AR-downregulated’ set includes *ABA1*, involved in ABA synthesis (Koornneef *et al.*, 1982), and the ‘AR-upregulated’ set includes *LPP2*, which negatively regulates ABA responsiveness (Katagiri *et al.*, 2005). It is possible, therefore, that this provides a molecular mechanism for AR-mediated processes, such that in imbibed seeds that have not undergone AR, accumulation of ABA1 transcripts results in the maintenance of dormancy through increased ABA synthesis, and in AR seeds, accumulation of LPP2 removes sensitivity of the seed to ABA, allowing completion of germination (Figure 6b). The 121 genes identified in the D > *aba1-1* F gene set (Table S1) represent downstream genes that are ABA responsive and may contribute to dormancy maintenance. Interestingly the ‘ABA-responsive’ gene *RD29B* is also part of the ‘AR-downregulated’ set, which may suggest that it is an early target of increased *ABA1* function (Figure 6b). Although the results presented here indicate that ABA is not a requirement for dry AR regulation of gene expression (Figures 1c and 2b), other ABA-associated processes (including seed dormancy and drought responses) are known to have both ABA-dependent and ABA-independent components. Therefore, we cannot rule out the possibility that there may be a small influence of ABA on AR, but the observation of similarities in transcriptomes between F and D WT and mutant seeds, and similarities in types of functional groups observed in these transcriptome datasets, argues strongly that ABA at most must have a very small role in dry seed AR, as opposed to its major role in dormancy of the imbibed seed.

The uncovering of AR-regulated gene sets has implications for the study of dormancy cycling in the soil seed bank, and for mechanisms influencing the domestication of crop plants via the removal of seed dormancy. Arabidopsis is a winter annual (Baskin and Baskin, 1998) that germinates mainly in autumn, and exposure to high summer temperatures leads to a loss of dormancy. If germination is prevented, seeds subsequently enter a dormancy cycling process that can last several years. As seeds in the soil can exist in degrees of hydrated states, those entering dormancy cycling in natural environments would need AR to occur in the subsequent unimbibed state to remove dormancy and allow germination to be completed. We have shown that secondary dormancy resets the expression of AR-regulated gene sets, and so it is possible that this resetting provides the mechanism to allow dormancy cycling to continue through multiple iterations in the soil, by preventing the completion of germination via the action of the ‘AR-downregulated’ gene set, which includes *ABA1* (Marin *et al.*, 1996) as well as genes encoding stress-related proteins [including several heat-shock proteins (Wehmeyer *et al.*, 1996), *ERD1* (Kiyosue *et al.*, 1993), *RD29B* (Uno *et al.*, 2000)] and several dormancy-associated genes (including *DRM1*; Stafstrom *et al.*, 1998). It is possible, therefore, that this provides a molecular explanation for AR-related mechanisms that regulate dormancy maintenance in seed populations cycling in the soil bank, as ABA synthesis and perception have been shown to be important in the maintenance of the imbibed dormant state (Koornneef *et al.*, 1989).

Loss of the capacity for seed dormancy is one of the major traits acted on by evolutionary processes during the introduction of wild plant species into agriculture (the ‘domestication syndrome’) (Harlan, 1992). There are two possible mechanisms that could lead to reduced seed dormancy in domesticated crop species: removal of the capacity for dormancy (for example by crippling the functions of genes such as *ABA1*) or reduction in the AR dependence of seeds. These are two distinct molecular processes occurring in two different developmental pathways. By defining gene sets that are AR regulated it may now be possible to distinguish between the contributions of these two mechanisms, for example through the identification of molecular markers or transcriptome developmental signatures that can be used to differentiate crop varieties with differing D/AR characteristics. As a lack of dormancy can be a major agronomic problem (for example in the disorder pre-harvest sprouting in wheat and barley), such approaches may provide the tools to deliver improved AR and dormancy characteristics (Gubler *et al.*, 2005; Holdsworth *et al.*, 1999; McKibbin *et al.*, 2002; Wilkinson *et al.*, 2002).

## Experimental procedures

### Plant genetic material

Wild-type accession Cvi and mutant seeds *fus3-8*, *rdo2* and *abi3-4* were provided by Prof. M. Koornneef (MPI, http://www.mpiz-koeln.mpg.de); L*er*, *abi1-1*, *aba1-1* were obtained from NASC (http://arabidopsis.info). All mutants are in the L*er* background.

### Plant growth and seed treatments

Arabidopsis WT and mutant plants were grown as previously described (Footitt *et al.*, 2006). Dry seeds were harvested from main bolt siliques and maintained under constant storage conditions (24°C in the dark). Germination assays were carried out on water agarose under constant light; germination was assessed as endosperm rupture by the elongating radicle over 7 days (Footitt *et al.*, 2006). The AR status of dry seed lots was determined by an assay of the germination potential of appropriate WT accessions. AR was considered to be complete following a time period that allowed WT accessions to achieve 100% germination after 7 days of imbibition. Freshly harvested seed lots were assayed when siliques were yellow, but not completely dehisced; stored seed lots of mutants were assayed following storage for time periods that at least matched those required for complete AR of WT accessions. Under the storage conditions used here, L*er* seeds completely after-ripen within 60 days (Figure 1a). All seed lots were dry stored in the same cabinet in the dark at 24°C in sealed glassine bags. Where appropriate, exogenous (+/–) ABA (Sigma-Aldrich, http://www.sigmaaldrich.com), was included in media at a concentration of 10 μm.

### Transcriptome and RNA analyses, and bioinformatic treatment of transcriptome datasets

All experiments analysing RNA expression levels were carried out using three replicates of seed materials, obtained from three independent seed lots, from plants grown under controlled-environment conditions. Total RNA was extracted from seed material and real-time quantitative PCR was carried out using the Universal ProbeLibrary system (Roche Diagnostics, http://www.roche.com), with primers and probes for each gene assay designed using the Universal ProbeLibrary Assay Design Center (Roche Applied Science) as previously described (Carrera *et al.*, 2007). Transcriptome analyses were carried out using the NASC Affymetrix ATH1 genechip service. All array information reported here is available online from NASC arrays (http://affymetrix.arabidopsis.info/narrays/experimentbrowse.pl). Gene expression levels were measured using the microarray suite 5.0 software (Affymetrix, http://www.affymetrix.com), and further analysis was performed with genespring 7.3 software (Agilent, http://www.agilent.com) using a twofold differential expression between samples (*P* value < 0.05 in a one-way anova) to define differentially regulated genes. Normalized expression values from microarray datasets were used to obtain ratios of expression. Developmental signatures were obtained using the TAGGIT workflow within Microsoft Excel (http://www.microsoft.com) (Carrera *et al.*, 2007).

## Supplementary Material

The following supplementary material is available for this article online:

Figure S1 Ratio of normalized expression of after-ripening (AR)-regulated and independent gene sets in wild-type (WT) and mutant seeds at 24 h of imbibition.

Figure S2 Gene expression in fresh (F) and stored (S) wild-type (WT) and mutant seeds.

Figure S3 Normalized expression of after-ripening (AR)-regulated and independent gene sets in embryo and endosperm material.

Figure S4 Influence of exogenous ABA and of primary and secondary dormancy status on expression characteristics of afterripening (AR)-regulated and independently regulated gene sets at 24 h of imbibition.

Table S1 Descriptions of differentially upregulated genes in comparisons used in this paper.

Table S2 After-ripening (AR)-regulated and independent gene sets.

Table S3 (A) Ratios of normalized expression and (B) statistical data of ratios of expression for gene expression comparisons used for the construction of box plots.

This material is available as part of the online article from http://www.blackwell-synergy.com
