# Supplementary material for: Seed after-ripening is a discrete developmental pathway associated with specific gene networks in Arabidopsis
Source: Plant J. 2008 Jan;53(2):214–24. doi: 10.1111/j.1365-313X.2007.03331.x (PMC2254144; doi:10.1111/j.1365-313X.2007.03331.x)
Supplement: Figure S1 — Ratio of normalized expression of after-ripening (AR)-regulated and independent gene sets in wild-type (WT) and mutant seeds at 24 h of imbibition. [file tpj0053-0214-sm-figs.pdf]

# Supplementary Figure 1

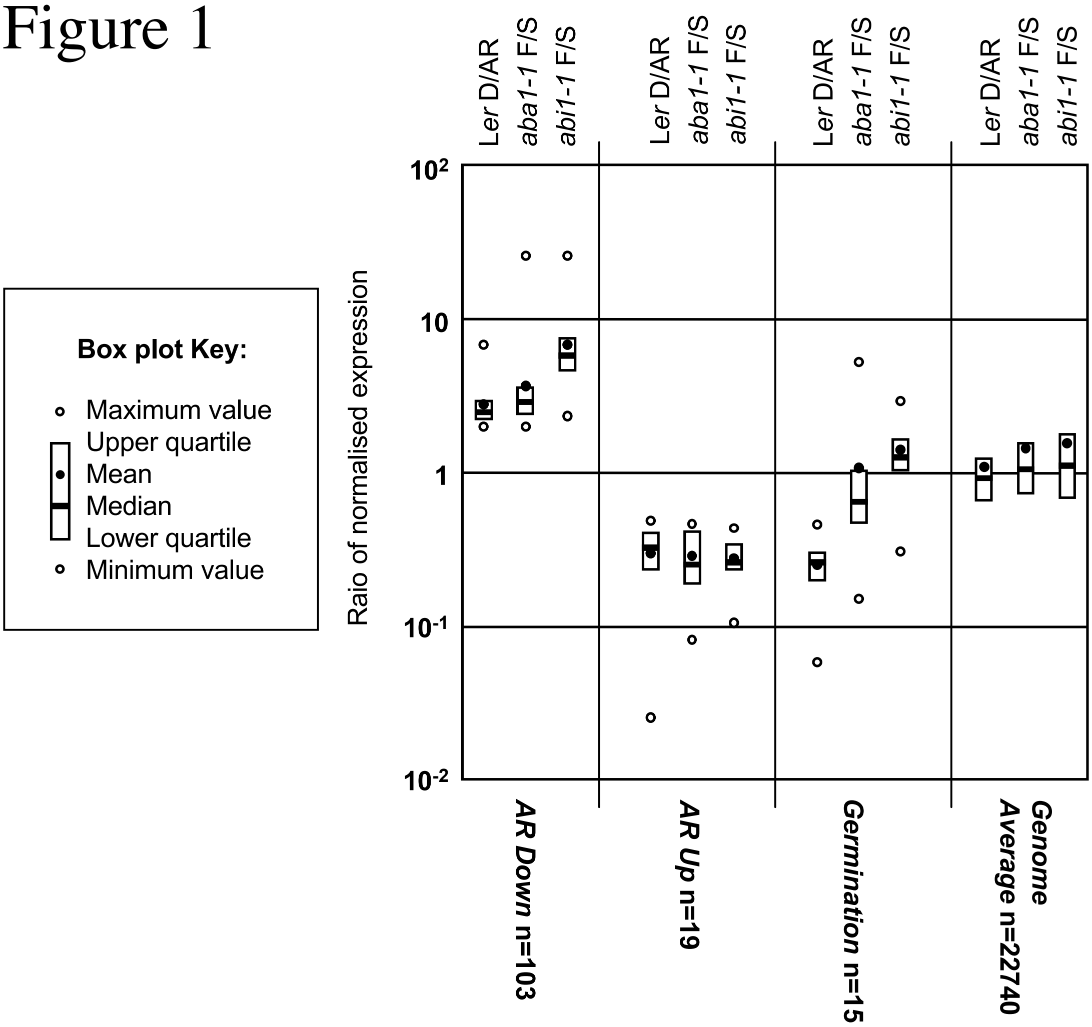


**Supp figure 1. Ratio of normalised expression of AR-regulated and independent gene sets in WT and mutant seeds at 24h imbibition.**
Ratio of normalised expression of the AR-'up', AR-'down' and germination-up gene sets in accession L*er* after-ripened and dormant samples, and *aba1-1* and *abi1-1* fresh and stored samples. In each case the ratio of normalised expression is plotted on a logarithmic scale.

# Supplementary Figure 2

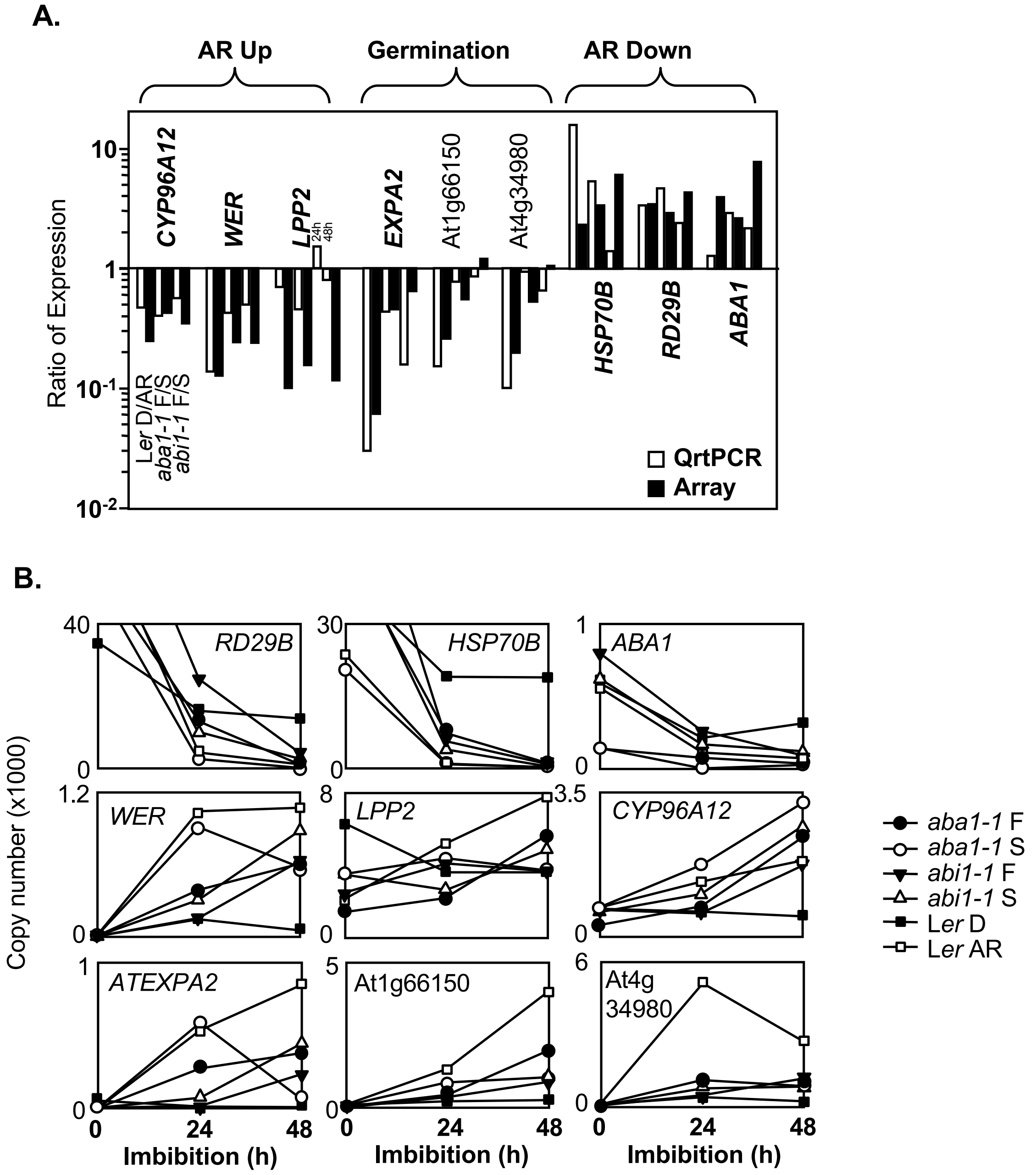


**Supp figure 2. Gene expression in Fresh and Stored WT and mutant seeds.**
A. Single time-point analysis ratio of expression of particular genes from the AR-'up' (*CYP96A12*, *WER*, *LPP2*), Germination (*ATEXPA2*, *At1g66150*, *At4g31500*) and AR-'down' gene sets (*RD29B*, *HSP70B*, *ABA1*) (indicated). Expression measured in L*er*, *aba1-1* and *abi1-1* 24h imbibed seeds derived from quantitative (Q)rtPCR or microarray analysis of RNA expression. For each gene comparisons are presented in the following order: L*er* D/AR, *aba1-1* F/S, *abi1-1* F/S. S, stored seeds, F, Freshly harvested, D, Dormant, AR After-Ripened. Both 24 and 48h data are presented for *LPP2 abi1-1*.
B. Quantitative rtPCR time-course analysis of expression of representative members of the AR-'up', AR-'down' and Germination-up gene sets over 48h imbibition on water agarose in constant light.
S, Stored seeds, F, Freshly harvested, D, Dormant, AR After-Ripened.

# Supplementary Figure 3

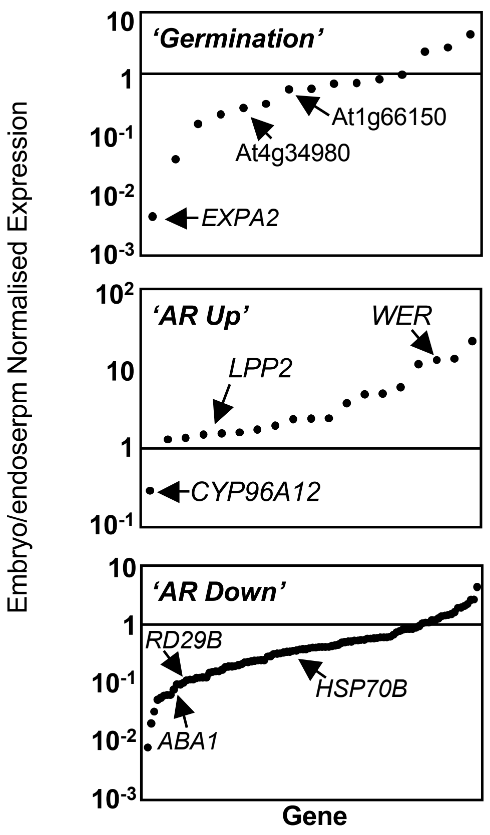


**Supp figure 3. Normalised expression of AR regulated and independent gene sets in embryo and endosperm material.**
Ratio of normalised expression of the AR-'up', AR-'down' and germination-up gene sets in accession Ler embryo or endosperm 1 d following transfer to 22°C after 3 d of cold stratification (Penfield *et al* 2006). Normalised expression values derived from microarray datasets are shown (Supp table 3). Ratios of expression of representative genes are indicated with arrows. In each case the ratio of normalised expression is plotted on a logarithmic scale.

# Supplementary Figure 4

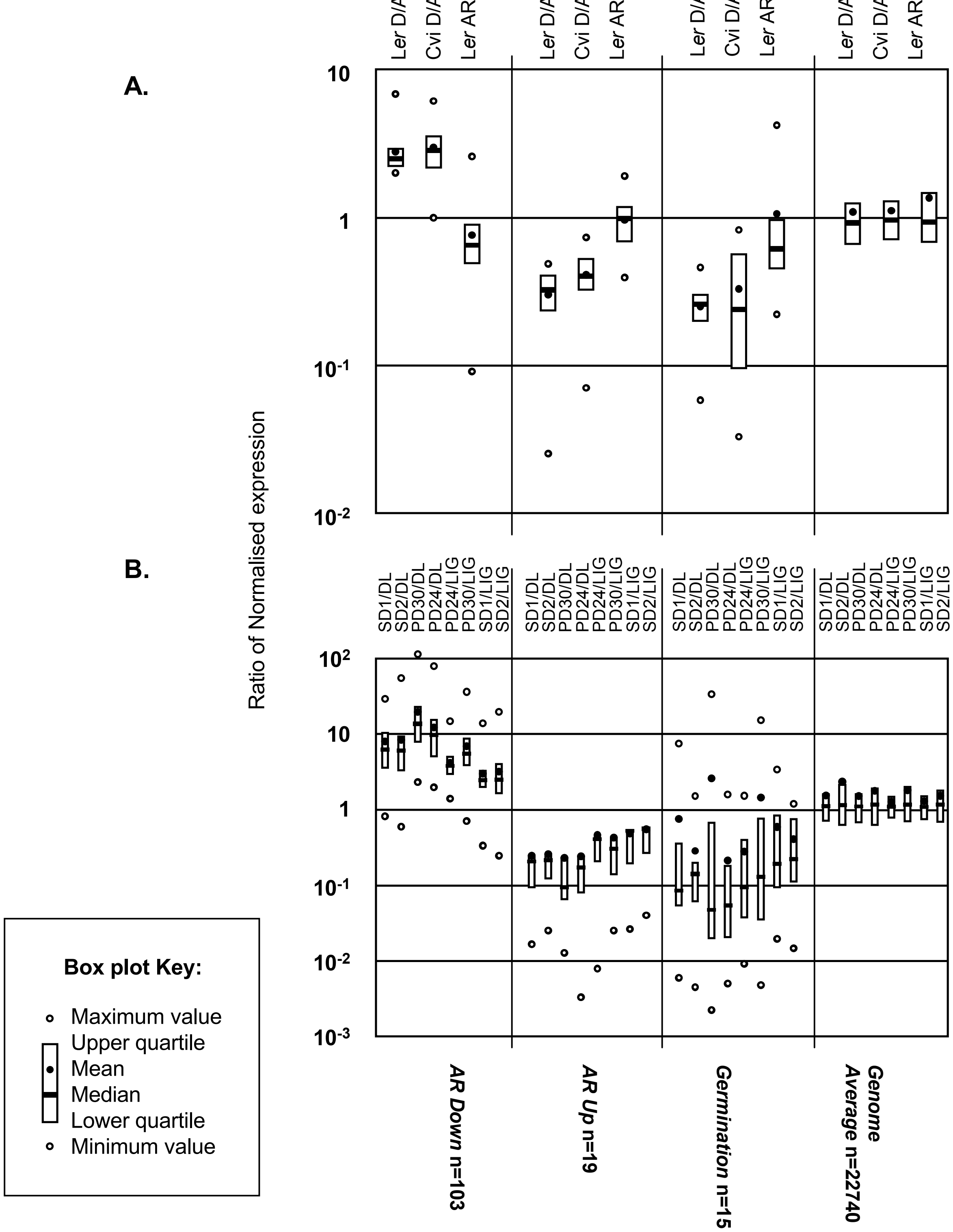


**Supp figure 4. Influence of exogenous ABA and of primary and secondary dormancy status on expression characteristics of AR-regulated and independently regulated gene sets at 24h imbibition.**
A. Boxplot showing ratio of expression of the AR-‘up’, AR-‘down’, ‘germination-up’ and genome average gene sets in Ler AR and D, Cvi AR and D and Ler AR imbibed on ABA (10 μM, AR+ABA). The ratio of normalised expression is plotted on a logarithmic scale.
B. Boxplot showing ratio of normalised expression of the AR-‘up’, AR-‘down’, ‘germination-up’ and genome average gene sets in accession Cvi after-ripened and dormant samples (Cadman *et al* 2006). For each gene set, data are presented for ratios for the following datasets: AR seeds imbibed in the dark subsequently given a red light treatment to promote germination (LIG), after-ripened seeds imbibed in the dark (DL), Primary Dormant 24h (PD24), Primary Dormant 30d (PD30), Secondary Dormancy state 1 (SD1), Secondary Dormancy state 2 (SD2). The ratio of normalised expression is plotted on a logarithmic scale.
