# Supplementary material for: Seed after-ripening is a discrete developmental pathway associated with specific gene networks in Arabidopsis
Source: Plant J. 2008 Jan;53(2):214–24. doi: 10.1111/j.1365-313X.2007.03331.x (PMC2254144; doi:10.1111/j.1365-313X.2007.03331.x)
Supplement: Supplementary file 5 [file tpj0053-0214-sm-legends.doc]

**Supplementary figure legends:**

**Supplementary table 1**. Descriptions of differentially up-regulated genes in comparisons used in this paper.

**Supplementary table 2**. AR-regulate and independent gene sets.

**Supplementary table 3**. (A) Ratios of normalized expression and (B) statistical data of ratios of expression for gene expression comparisons used for the construction of boxplots.

Supplementary figure 1. Ratio of normalised expression of AR-regulated and independent gene sets in WT and mutant seeds at 24h imbibition.

**Supplementary figure 2.** Gene expression in Fresh and Stored WT and mutant seeds.

Supplementary figure 3. Normalised expression of AR regulated and independent gene sets in embryo and endosperm material.

**Supplementary figure 4.** Influence of exogenous ABA and of primary and secondary dormancy status on expression characteristics of AR-regulated and independently regulated gene sets at 24h imbibition.
